# Characterization and phylogenetic analysis of the complete mitochondrial genome of the firemouth cichlid, *Thorichthys meeki* (Perciformes: Cichlidae)

**DOI:** 10.1080/23802359.2022.2086080

**Published:** 2022-06-30

**Authors:** Sang-Eun Nam, Jae-Sung Rhee

**Affiliations:** a Department of Marine Science, College of Natural Sciences, Incheon National University, Incheon, South Korea; b Research Institute of Basic Sciences, Incheon National University, Incheon, South Korea; c Yellow Sea Research Institute, Incheon, South Korea

**Keywords:** Complete mitogenome, cichlid fish, *Thorichthys meeki*, phylogenetic analysis

## Abstract

Here, we report information on the complete mitochondrial genome of the firemouth cichlid, *Thorichthys meeki* (Brind 1918). Illumina HiSeq genome sequencing produced the assembly of a circular mitogenome of 16,527 base pairs (bp) from *T. meeki* consisting of 46.8% GC nucleotides, 13 protein-coding genes (PCGs), two ribosomal RNA (rRNA) genes, 22 transfer RNA (tRNA) genes, and a putative control region as shown in the typical teleost gene composition. The gene order of the *T. meeki* mitogenome was identical to that of other cichlid species. A maximum likelihood phylogenetic tree based on mitochondrial PCGs showed a close relationship of *T. meeki* with *Thorichthys aureus* (Gunther 1862) within Heroini tribe.

One of the most species-rich clades in spiny-rayed fish, the family Cichlidae has a wide distribution range with abundance and diversity in Africa, South America, and Central America (Kornfield and Smith 2000; Smith et al. 2008; Nelson et al. 2016). Of cichlids, the subfamily Cichlasomatinae is divided into two tribes, the Cichlasomatini (Swainson 1839) and the Heroini (Kullander 1998). Approximately 150 species involved in the tribe Heroini are mainly distributed in Middle America with high morphological and ecological diversity (Matamoros et al. 2015; Arbour and López-Fernández 2016). Phylogenetic relationships of heroine cichlids have been consistently studied using morphological and molecular parameters (Concheiro Pérez et al. 2007; McMahan et al. 2015; Říčan et al. 2016). Although the reciprocal monophyly of the major members in the tribe Heroini is well-established, many areas of the phylogenetic relationship are still poorly resolved in this tribe because of biogeographic discordance in Middle and South American Heroini and limited genomic information (Říčan et al. 2016; Tagliacollo et al. 2017; Ilves et al. 2018; Alda et al. 2021). Thus, comprehensive mitogenomic information on heroine cichlids would be useful in establishing their appropriate phylogenetic placement and understanding biogeographic reconstruction in the tribe Heroini.

*Thorichthys meeki* (Brind 1918), referred to as a firemouth, is a Mesoamerican cichlid. The firemouth cichlid is a small and popular ornamental freshwater fish because of various of color morphs such as brilliant red–orange ventral coloration and easy handling. A specimen of *T. meeki* was collected from Lake Osborne (26°35′N, 80°04′W), Florida, USA. All samples, including total DNA and specimen, were deposited at the Research Institute of Basic Sciences of Incheon National University (Specimen ID: 2014-Cichlidae-03; https://www.inu.ac.kr/user/indexMain.do?siteId=ribs) by Dr. Sang-Eun Nam (se_nam2@inu.ac.kr). Detailed descriptions for all materials and methods of total genomic DNA extraction from muscle tissue, library construction with the TruSeq DNA Sample Preparation Kit (Illumina, San Diego, CA, USA) for Illumina HiSeq sequencing (150 bp; HiSeq X ten), and assembly are omitted as described in our previous study (Nam et al. 2022). After the quality check process, 31,736,328 filtered reads were obtained from 42,011,300 raw reads. Finally, *de novo* assembly was conducted with various k-mers using SPAdes version 3.13.0 (Bankevich et al. 2012) with default parameters and a circular contig of the *T. meeki* mitogenome was obtained. The resulting contig consensus sequence was annotated using MITOS2 (Bernt et al. 2013) and tRNAscan-SE 2.0 (Lowe and Eddy 1997). BLAST analysis confirmed the identity of each gene (http://blast.ncbi.nlm.nih.gov).

The nucleotide composition of the *T. meeki* circular 16,527 bp mitogenome (GenBank accession no. MZ427899) was 28.0% A, 31.3% C, 15.5% G, and 25.2% T. The gene order and composition of the *T. meeki* mitogenome were identical to those of other mitogenomes of the tribe Heroini. A phylogenetic tree was constructed using the concatenated set of all 13 PCGs of the *T. meeki* mitogenome, 77 published complete mitogenomes of cichlids, and an outgroup from the family Balistidae (Figure 1). The phylogenetic analysis was performed using the maximum likelihood method and GTR + G + I model with a bootstrap of 1000 replicates. The overall topology of each tribe was consistent with previous phylogenetic results, as the members of the tribe Heroini are clearly separated from the members of the tribe Cichlasomatini (Matschiner et al. 2017; Alda et al. 2021) and are also supported by morphological parameters (Smith et al. 2008). *T. meeki* grouped together with other representatives of the tribe Heroini. Of the tribe Heroini, *T. meeki* is closely related to the member of the same genus, *Thorichthys aureus* (NC_031182).

**Figure 1.**
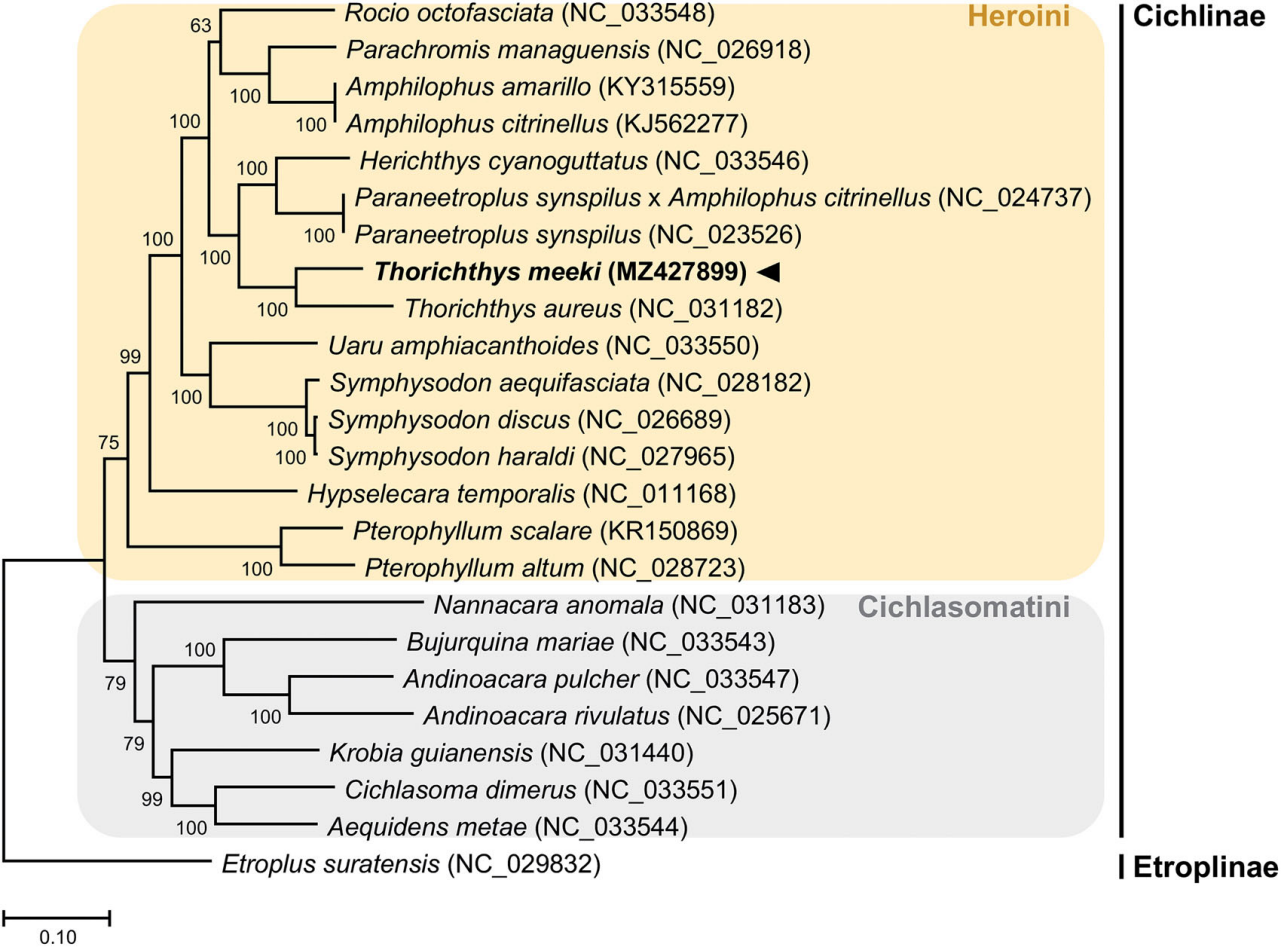
Maximum likelihood (ML) phylogeny of 78 published complete mitogenomes of cichlids and an outgroup from the family Balistidae based on the concatenated nucleotide sequences of protein-coding genes (PCGs). The phylogenetic analysis was performed using the maximum likelihood method and GTR + G + I model with a bootstrap of 1000 replicates. Numbers on the branches indicate ML bootstrap percentages. DDBJ/EMBL/Genbank accession numbers for published sequences are incorporated. The black triangle indicates the cichlid analyzed in this study.

## Author contributions

S.-E. Nam: conceptualization, methodology, software, and writing; J.-S. Rhee: conceptualization, supervision, reviewing, and editing; All the authors agreed to be accountable for all aspects of the work.

## Data Availability

BioProject, BioSample, and SRA accession numbers are https://www.ncbi.nlm.ni h.gov/bioproject/PRJNA743754, https://www.ncbi.nlm.nih.gov/biosample/SAMN20059977, and https://www.ncbi.nlm.nih.gov/sra/?term=SRR15356124, respectively. The data that support the findings of this study are available at the National Center for Biotechnology Information (NCBI) at https://www.ncbi.nlm.nih.gov, with the accession number MZ427899.
